# HEART Score of Four for Age and Risk Factors: A Case Series

**DOI:** 10.7759/cureus.9576

**Published:** 2020-08-05

**Authors:** James L Webb, Matthew Streitz, Jessica Hyams, Michael April, Joshua J Oliver

**Affiliations:** 1 Emergency Department, San Antonio Uniformed Services Health Education Consortium, San Antonio, USA

**Keywords:** heart score, chest pain, emergency department, four, risk factors, age, acute coronary syndrome, risk stratify, major adverse cardiac events

## Abstract

Chest pain is a frequent chief complaint in the ED. Identifying acute coronary syndrome (ACS) and establishing proper disposition for further risk assessment for major adverse cardiac events are paramount. The HEART Score is a key decision-making tool used to determine patient risk and disposition. One scenario with a potential drawback of the HEART Score is found in patients with a score of four based solely on age and risk factors. The HEART Score categorizes a score of three or less as low risk, and patients with scores above this threshold are typically admitted. We present six cases of chest pain presenting to a military emergency department with a score of four based solely on age and risk factors. They represent every such case found in a previously created database used to validate the HEART Score. We followed each case forward one year in electronic medical records to identify major adverse cardiac events. With the exception of one case that was placed on hospice for non-cardiac reasons and subsequently lost to follow up, there were no adverse events. There is a rising concern for increasing hospital admission rates, overuse of resources, and cost. We highlight that this subset of HEART Score patients requires a more nuanced risk stratification in the ED. It may be worth the time and effort to risk stratify this subset with coronary computed tomography angiography. This additional effort may help reduce admission at such a patient’s current and future presentations to the ED for chest pain.

## Introduction

Chest pain is one of the most common chief complaints in the ED accounting for almost eight million visits annually and more than 5% of all visits [1,2]. It is essential for ED physicians to determine which cases are acute life-threatening conditions such as acute coronary syndrome (ACS). ED physicians are commonly tasked with determining if further evaluation is required for risk stratification either though admission or outpatient follow-up. In doing so, they must consider the patient’s acute risk of developing an adverse outcome.

The American Heart Association states that it is reasonable for low-risk patients to be discharged with close follow-up and risk stratification testing such as myocardial perfusion imaging or exercise stress testing. They also recommend admission for inpatient evaluation and management for patients with a concerning picture for ACS; specifically, in those with recurrent symptoms, a concerning electrocardiogram (ECG), or elevated troponin. It is important for ED physicians to determine which cases require admission and which are safe for discharge [2,3].

A valuable decision-making tool to help disposition patients with symptoms concerning for ACS is the HEART Score [4-6]. Unlike other scoring systems like the thrombolysis in myocardial infarction (TIMI) score or the Global Registry of Acute Coronary Events (GRACE) Score, the HEART Score has been developed and validated in the ED setting [4-6]. Multiple external validation studies and reviews have shown its safety and accuracy in predicting the acute risk of major adverse cardiac events (MACE) [7-9].

The HEART Score uses five components, which are assigned a score of 0, 1, or 2 points each (Table 1) [4-6,10]. These components are History (H), ECG (E), Age (A), Risk Factors (R), and Troponin (T). Combined, a score between 0 and 3 is considered low-risk for MACE which was defined as myocardial infarction (MI), revascularization, or death [4]. Those at low-risk are largely considered safe to be discharged for further outpatient risk stratification. A score of four or more indicates the patient is not low-risk and requires admission for observation and/or intervention [4-6]. The HEART Pathway has subsequently been developed which utilized a second troponin three hours later. This resulted in an overall decrease length of stay and showed risk of MACE to be <1% in those with a HEART Score of three or less [11-14].

While the HEART Score is a useful tool, a scenario that many Emergency Medicine physicians know to be frustrating for their Internal Medicine colleagues is when patients present to the ED with chest pain and a resulting HEART Score of four based entirely on the age and risk factors. The frustration comes from the fact that these characteristics are static. If strictly applied, the HEART Score would recommend these patients be admitted upon every ED presentation for chest pain [4].

In order to explore the question of risk for MACE in patients with a HEART Score of four based solely on their age and risk factors, we evaluate a case series of six patients. These six cases were not chosen at random. They were taken from a database previously created as part of a chart review study conducted at a military tertiary care center’s ED which the purpose was to externally validate both the HEART Score and HEART Pathway among a population of Active Duty Military, Retirees, and their dependents [8,12,13]. Data were collected on 625 subjects who presented for chest pain during the study period in 2016. Of the 449 that were included, the six subjects in this series are the only ones with a HEART Score of four based solely on age and risk factors [12]. Via chart review, we followed each subject out one year from their initial ED presentation to observe for MACE occurrences. To the best of our knowledge, no other work has been developed on this subset of patients. The initial HEART Score studies followed subjects for several weeks to assess for MACE occurrences. We followed these patients for one year in the hope that a trend might present itself that would allow us to identify which subjects could be dispositioned from the ED.

## Case presentation

Case 1

A 65-year-old female with a history of diabetes, hypertension, hyperlipidemia, and obesity was transferred from an urgent care center and presented with four days of chest pain. She described her pain as a 4/10 central chest and epigastric pain that felt like she needed to belch. Her symptoms had been constant, non-exertional, and non-radiating. She denied shortness of breath, diaphoresis, nausea or vomiting. She noted tingling in her lips; with review of symptoms (ROS) otherwise negative. Vital signs were within normal limits. Physical exam noted tenderness to palpation of mid chest, reproducing her pain. ECG demonstrated normal sinus rhythm without signs of ischemia. The physician reviewed a nuclear medicine myocardial perfusion imaging report from seven years prior which was unremarkable and showed normal left ventricular function. Initial and repeat troponin were both <0.01 ng/mL. This gave the patient a HEART Score of History(H) 0, ECG(E) 0, Age(A) 2, Risk(R) 2, Troponin(T) 0, for a total of 4 suggesting admission. 324 mg of oral aspirin was given along with 2 mg intravenous morphine and 4 mg intravenous ondansetron`(Table 1). She was diagnosed with atypical chest pain and discharged with strict return precautions and primary care follow-up for further risk stratification.

**Table 1:** The Heart Score ECG: electrocardiogram a: Risk factors: hypertension, diabetes mellitus, hypercholesterolemia, smoker, family history, and obesity b: Atherosclerotic disease: history of coronary revascularization, myocardial infarction, stroke, or peripheral vascular disease

Component	Grade	Score
History	Highly suspicious	2
	Moderately suspicious	1
	Slightly or non-suspicious	0
ECG	Significant ST depression	2
	Nonspecific repolarization disturbance	1
	Normal	0
Age	≥ 65 years	2
	45 – 64 years	1
	< 45 years	0
Risk Factors	≥ 3 risk factors^a^ or history of atherosclerotic disease^b^	2
	1 – 2 risk factors	1
	No known risk factors	0
Troponin	≥ 3x normal limit	2
	> 1 to < 3x normal limit	1
	≤​​​​​​​​​​ normal limit	0

The patient followed-up three days later. The primary care physician (PCP) noted the patient had a history of gastroesophageal reflux disease (GERD) with a hiatal hernia and the pain was similar to the patient’s prior GERD symptoms. The PCP stated there were no red flags during the follow-up visit. She was diagnosed with GERD without esophagitis and given a trial of a proton-pump inhibitor (PPI). On subsequent primary care follow-up, the symptoms had resolved. Over the course of the following year, no further cardiac risk stratification studies were noted to be performed. After one year since the ED visit, the patient had no occurrence of MACE.

Case 2

A 73-year-old female with a history of hypertension, diabetes, and obesity presented with one day of chest pain. She described it as sudden-onset while at rest with a stabbing sensation radiating to her left shoulder. Other than a cough, the remainder of her ROS was negative. She was given 0.4 mg sublingual nitroglycerine without relief, once by Emergency Medical Services and again in the ED. She received 4 mg intravenous morphine with improvement of symptoms. Vital signs, physical exam, chest x-ray, and basic lab work were unremarkable including a troponin level <0.010 ng/mL. ECG showed normal sinus rhythm with no evidence of ischemia. She was diagnosed with chest pain and admitted to Internal Medicine with a HEART Score of 4 (H0E0A2R2T0). 324 mg oral aspirin was given. 

**Figure 1:**
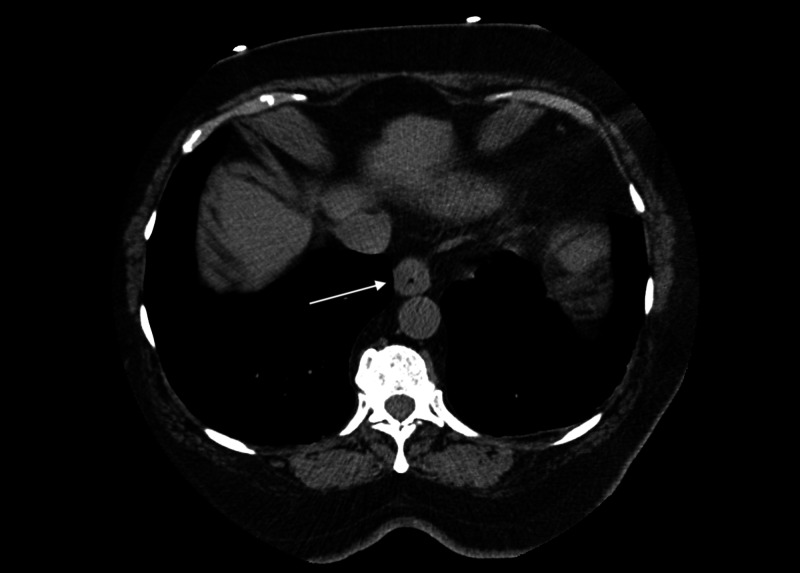
Non-contrasted computed tomography noting circumferential thickening of distal esophagus (arrow)

During her inpatient stay, serial troponin labs were negative. The patient was evaluated by Cardiology and a nuclear medicine myocardial perfusion study was performed showing no ischemia and normal left ventricular function. A prior chest CT noted esophageal changes consistent with GERD and, in the setting of recently starting clindamycin for a foot infection, she was started on ranitidine for possible gastric reflux as the etiology of her chest pain (Figure 1). The inpatient service also noted position-dependent pain concerning musculoskeletal pain so a topical lidocaine patch was prescribed. She was diagnosed with non-cardiac chest pain and discharged with primary care follow-up.

**Figure 2:**
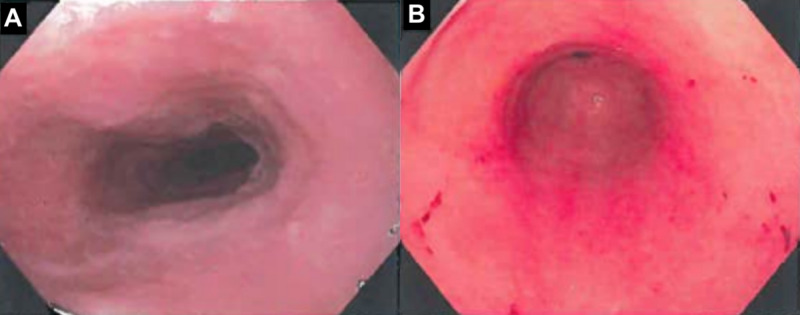
Esophagogastroduodenoscopy noting normal appearing proximal esophagus (A) and erythematous mucosa and heme in antrum/body of the stomach (B) consistent with gastritis

She returned to the ED one month later with mid-to-left sided pressure/sharp chest pain without relief from nitroglycerin. ECG and cardiac biomarkers were unremarkable. The patient was admitted with gastroenterology (GI) consult and an esophagogastroduodenoscopy (EGD) was performed noting gastritis (Figure 2). EGD biopsy would eventually showed no evidence of H. pylori. GI’s leading diagnosis for the patient’s symptoms was secondary to inactive gastritis. The patient was started on a PPI with PCP follow-up.

The patient would be admitted several other times over the following year: at six weeks from initial presentation (diagnosed with non-cardiac chest pain, pain syndrome suspect fibromyalgia), at two months (diagnosed with non-cardiac chest pain), and at six months (diagnosed with non-cardiac chest pain, left shoulder/arm pain). The patient had negative troponins throughout these admissions. Cardiology was consulted during multiple admissions without further concern to risk stratify with imaging studies. After one year, the patient was without evidence of MACE in the medical record.

Case 3

An 87-year-old male with a history of hypertension, hyperlipidemia, and hemorrhagic stroke presented with one hour of chest pain. He described the pain as 4/10, left-sided, and “pulsing”. The symptoms lasted for five minutes and resolved prior to arrival. The remainder of his ROS was negative. Two ECGs were performed noting non-ischemic sinus bradycardia. An initial and repeat troponin were both <0.010 ng/mL. The patient received 324 mg oral aspirin and was diagnosed with chest pain with a HEART Score of 4 (H0E0A2R2T0). The patient and his family opted for outpatient workup and management rather than be admitted. The patient was placed on an established accelerated diagnostic outpatient pathway with close follow-up for further cardiac risk stratification. 

**Figure 3:**
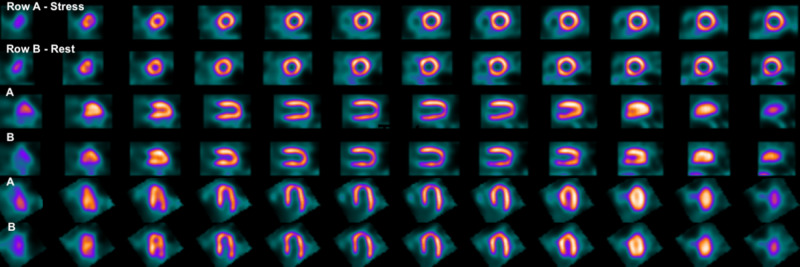
Nuclear medicine myocardial perfusion study with stress imaging (A) and rest imaging (B) noting no significant perfusion defects

He followed-up four days later and an outpatient nuclear medicine myocardial perfusion study was later performed which yielded no myocardial ischemia and normal left ventricular function (Figure 3). Within the following year, the patient received cardiac evaluation prior to surgery for elective total knee arthroplasty. He was found to be safe for surgery without further cardiac stress testing, imaging, or angiography. On routine primary care follow-up one year after the ED visit, the patient had no noted episodes of MACE.

Case 4

**Figure 4:**
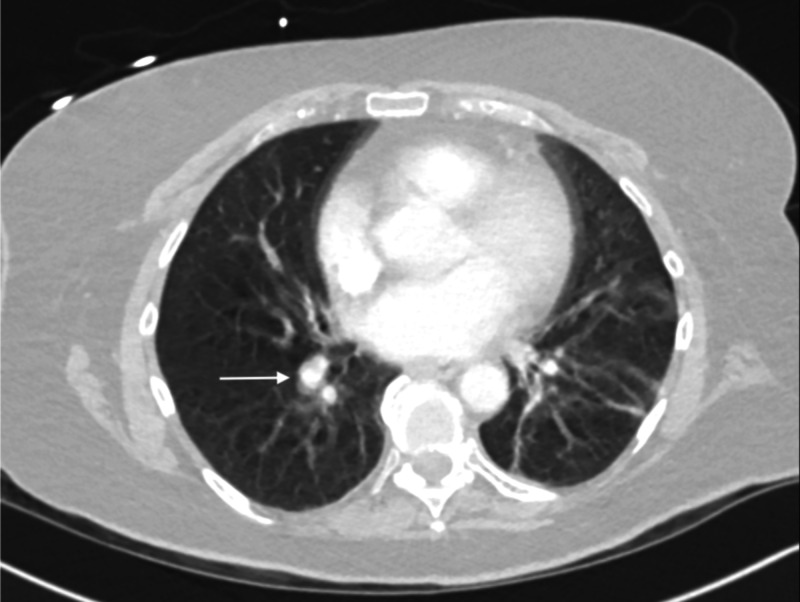
Computed tomography angiogram noting filing defect along the right lower lobe pulmonary artery walls (arrow) consistent with chronic pulmonary embolism appearance

A 73-year-old female with a history of coronary artery disease (CAD) with percutaneous coronary intervention two years prior, diabetes, hypertension, liver transplant (six years prior), and pulmonary embolism (PE) presented with diffuse 10/10 chest pain and upper abdominal pain for five hours. She reported nausea and vomiting, but denied shortness of breath. She had subjective fevers at home. The provider noted that the patient was in moderate distress and anxious on arrival. Vital signs were significant for blood pressure of 194/59 mmHg, but otherwise within normal limits and afebrile. ECG showed normal sinus rhythm without evidence of ischemia. Labs were remarkable for elevated liver function tests (LFTs) (total bilirubin 2.1 mg/dL, aspartate aminotransferase 407 U/L, alanine aminotransferase 303 IU/L, alkaline phosphatase 509 IU/L), creatinine 1.9 mg/dL, and lactate 3.3 mmol/L. Troponin was <0.010 ng/mL. No acute findings were noted on chest x-ray. CT noted bilateral segmental pulmonary embolisms, likely chronic (Figure 4). The patient was started on antibiotics. She was diagnosed with chest pain, sepsis with concern for ascending cholangitis, and bilateral PEs. Medical decision-making notes concern for ACS. She had a HEART Score of 4 (H0E0A2R2T0) and 324 mg oral aspirin was given.

She was admitted to the intensive care unit (ICU). Anticoagulation was held in the setting of prior life-threatening bleed and clinical doubt for acute PEs. GI was consulted whom suspected likely viral illness/gastroenteritis/hepatitis versus drug-induced hepatitis and magnetic resonance cholangiopancreatography was unremarkable. Her hospital course was complicated by a urinary tract infection and pneumonia. The patient was eventually discharged to rehab and given primary care follow-up.

Three months after the presentation, the patient had a routine follow-up. She noted to have continued episodic chest discomfort. She was diagnosed with atypical chest pain. A nuclear medicine study was schedule, but after discussing with her Cardiologist, no further risk stratification studies were completed. 

Just under one year from the initial ED visit, she was admitted for pyelonephritis and central line infection. Upon discharge, the patient moved out-of-state to live with her daughter given she was unable to care for herself. Case Management followed up with the patient, which was one year after her initial visit. She had established a new PCP and, apart from one episode of urinary urgency, she reported no new or acute medical issues and there was no evidence of MACE.

Case 5

**Figure 5:**
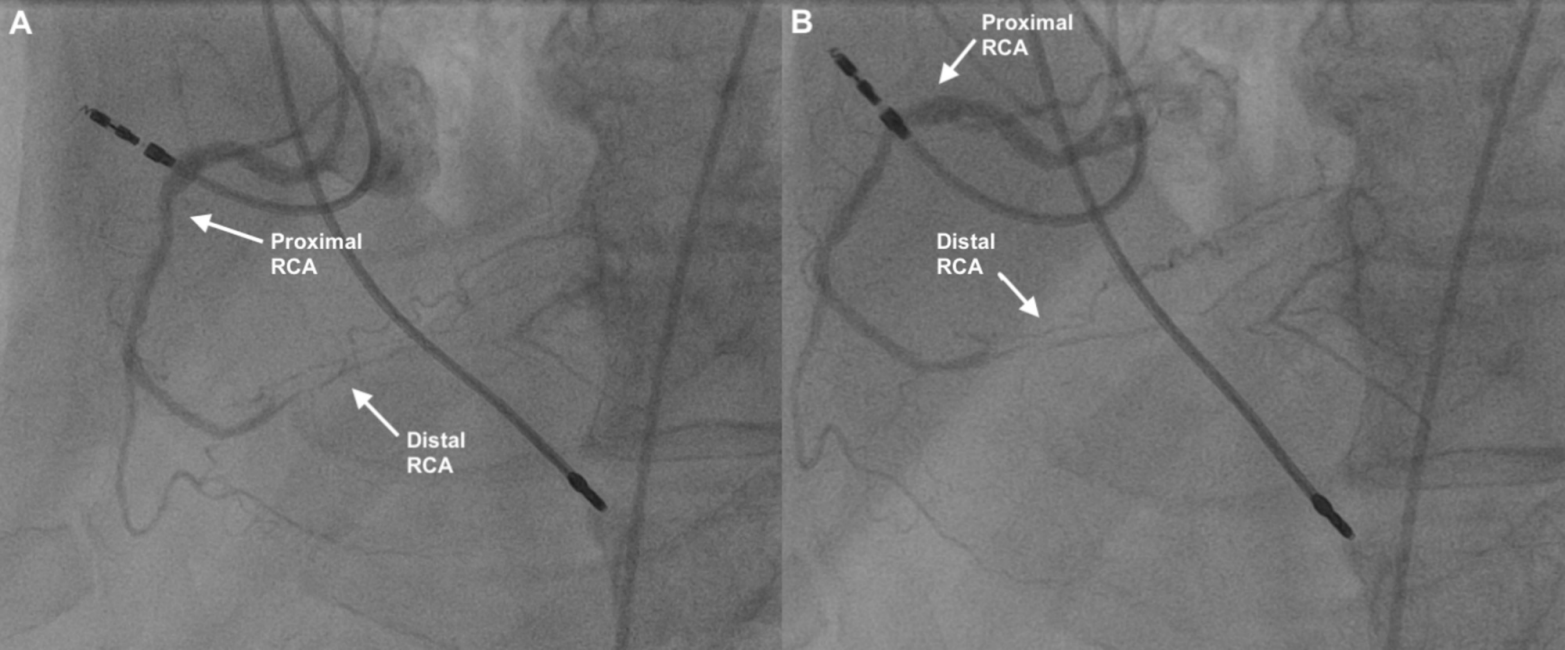
Two images (A and B) from cardiac catheterization of the right coronary artery from two years prior to presentation noting diffuse stenosis of the distal right coronary artery RCA = right coronary artery

A 79-year-old military retiree with a history of CAD and hypertension presented with one hour of chest pain that woke him up from sleep. He had a history of MI two years prior. At that time, medical therapy was pursued given the patient had poor anatomical targets for revascularization (Figure 5). However, both aspirin and clopidogrel had since been discontinued due to significant gastrointestinal bleeding secondary to a colon tumor and subsequent colectomy. He continued to be on a beta-blocker, a statin, and a daily nitroglycerin patch. On this ED visit, the pain was described as a pressure that was initially severe, reduced to a 2-3/10, and was non-radiating. ROS was unremarkable. Troponin was <0.01 ng/mL. ECG showed sinus rhythm with left ventricular hypertrophy, but no evidence of ischemia. The patient was admitted with a HEART Score of 4 (H0E0A2R2T0). Serial troponins were negative. Cardiology was consulted noting the pain was reproducible and inconsistent with ischemic chest pain. They did recommend an inpatient risk stratification study, but the patient elected to be discharged home with outpatient cardiology follow-up. He remained asymptomatic through hospital admission and diagnosed with atypical chest pain.

He followed up with cardiology without further concern or changes in medications and no further imaging was done. Four months later, he was placed on hospice for advanced cholangiocarcinoma. No MACE was noted within that time. Although he does not appear in any death registry, we assume he passed. Given the lack of documentation after being placed on hospice and his complicated medical problems, it is difficult to determine what he may have died of.

Case 6

**Figure 6:**
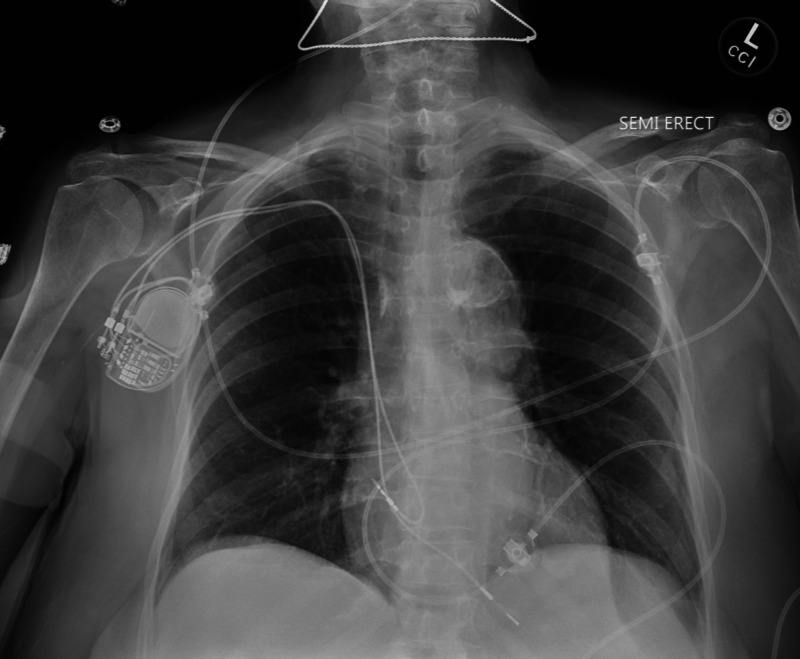
Chest x-ray without evidence of acute cardiopulmonary abnormality. Note image without evidence of significant pulmonary edema

A 92-year-old female with a history of second-degree type II heart block with dual-chamber pacemaker, hypertension, hyperlipidemia, and ischemic stroke presented with five hours of chest pain. It was described as 5/10 epigastric/chest tightness that started at rest and resolved prior to arrival. Symptoms were non-radiating and without shortness of breath. The remainder of the reactive oxygen species (ROS) were negative. Initial vital signs showed elevated blood pressure of 252/95 mmHg, but were otherwise unremarkable. ECG noted atrial-paced, right bundle branch block without signs of ischemia. Serial troponins were <0.010 ng/mL and chest x-ray was normal (Figure 6). Lab work was otherwise unremarkable to include complete blood count, metabolic panel, and urinalysis. She was given 324 mg oral aspirin and started on an intravenous nicardipine drip. She was admitted to the ICU with the diagnosis of hypertensive emergency and chest pain with a HEART Score of 4 (H0E0A2R2T0).

During the ICU stay, the admitting service noted that the patient had been non-compliant with prescribed anti-hypertensive medications. The chest pain was presumed to be musculoskeletal and non-cardiac - stating it was reproducible, lasted seconds, and consistent with muscle spasm. Serial troponins were negative. A follow-up ECG was without ischemic changes. The patient was discharged home two days after admission with primary care follow-up. No cardiac risk stratification studies were performed during admission. The patient did return one month later with elevated blood pressure and inpatient adjustments were made to anti-hypertensive medications. No reported chest pain occurred at the return visit. On routine Cardiology follow-up for the patient’s pacemaker, one year after the initial ED visit, no new risk stratification testing was noted to be performed and no occurrences of MACE.

## Discussion

We present several cases of chest pain with a HEART Score of four, which is defined as an intermediate risk [4]. If strictly followed, all would have been admitted for observation and inpatient risk stratification testing [4]. In the ED along with inpatient and outpatient follow-up services, it appears clinical consideration was given towards each individual case when considering further cardiac workup and risk stratification. In two of the cases (Case 4 and Case 6), the patients would likely have been admitted regardless (for sepsis and hypertensive emergency, respectively). In multiple cases, patients were discharged from the ED and/or did not require risk stratification studies. These patients were evaluated for ACS and risk stratified in a variety of modalities, though their HEART Scores were identical.

Apart from one case in which the patient was placed on hospice, no confirmed MACE was observed within one year of initial presentation. Though we only present a handful of cases, the growing frequency of chest pain cases generates a rising concern for overcrowding, overuse or inappropriate use of resources, and increase cost [1,2,15]. The American Heart Association suggests the use of coronary computed tomographic angiography (CCTA) to assess patients with possible ACS with normal ECG, troponins, and without history of CAD [3]. Due to the lack of availability of technicians and cardiologists to perform and interpret this test, our facility does not routinely obtain CCTAs. However, studies have shown that the use of CCTA in low-to-intermediate risk patients leads to a safe and quicker discharge from the ED in many patients that would be admitted otherwise, and a negative imaging study resulted in a <1% risk of MACE in the first year of testing [16,17].There are limitations to CCTAs including the use of rate-lowering agents and appropriate renal function which may be an issue throughout this population with advanced age and medical problems. If available, however, this is a possible solution that would improve the length of stay and potentially limit re-admission for similar symptoms for months after imaging results.

## Conclusions

These cases highlight the need for a more in-depth evaluation of subsets of patients when applying the HEART Score such as patients with a score of four due to age and risk factors alone. Although the HEART Score is an important tool to rapidly assess patients with concern for ACS, there is a need for further investigation on the risk of MACE in certain subsets of patients rather than grouped together with a broad total HEART Score. There is also a need for potential consideration of alternative methods for ACS assessment and risk stratification that can decrease admission rates, resources, and cost.

## References

[1] Center for Disease Control and Prevention (2019). National hospital ambulatory medical care survey: 2016 emergency department summary tables. https://www.cdc.gov/nchs/data/nhamcs/web_tables/2016_ed_web_tables.pdf.

[2] Amsterdam EA, Kirk JD, Bluemke DA (2010). Testing of low-risk patients presenting to the emergency department with chest pain: a scientific statement from the American Heart Association. Circulation.

[3] Amsterdam EA, Wenger NK, Brindis RG (2014). 2014 AHA/ACC Guideline for the management of patients with Non-ST-Elevation Acute Coronary Syndromes: a report of the American College of Cardiology/American Heart Association Task Force on Practice Guidelines. J Am Coll Cardiol.

[4] Six AJ, Backus BE, Kelder JC (2008). Chest pain in the emergency room: value of the HEART score. Netherlands Heart J.

[5] Backus BE, Six AJ, Kelder JC (2010). Chest pain in the emergency room: a multicenter validation of the HEART score. Crit Pathw Cardiol.

[6] Backus BE, Six AJ, Kelder JC (2013). A prospective validation of the HEART score for chest pain patients at the emergency department. Int J Cardiol.

[7] Poldervaart JM, Reitsma JB, Backus BE (2017). Effect of using the HEART score in patients with chest pain in the emergency department: a stepped-wedge, cluster randomized trial. Ann Intern Med.

[8] Streitz MJ, Oliver JJ, Hyams JM (2018). A retrospective external validation study of the HEART score among patients presenting to the emergency department with chest pain. Intern Emerg Med.

[9] Fernando SM, Tran A, Cheng W (2019). Prognostic accuracy of the HEART score for prediction of major adverse cardiac events in patients presenting with chest pain: a systematic review and meta-analysis. Acad Emerg Med.

[10] Poldervaart JM, Reitsma JB, Koffijberg H (2013). The impact of the HEART risk score in the early assessment of patients with acute chest pain: design of a stepped wedge, cluster randomised trial. BMC Cardiovasc Disord.

[11] Mahler SA, Riley RF, Hiestand BC (2015). The HEART pathway randomized trial: identifying emergency department patients with acute chest pain for early discharge. Circ Cardiovasc Qual Outcomes.

[12] Oliver JJ, Streitz MJ, Hyams JM (2018). An external validation of the HEART pathway among emergency department patients with chest pain. Intern Emerg Med.

[13] Hyams JM, Streitz MJ, Oliver JJ (2018). Impact of the HEART pathway on admission rates for emergency department patients with chest pain: an external clinical validation study. J Emerg Med.

[14] Mahler SA, Lenoir KM, Wells BJ (2018). Safely identifying emergency department patients with acute chest pain for early discharge. Circulation.

[15] Brooker JA, Hastings JW, Major-Monfried H (2015). The association between medicolegal and professional concerns and chest pain admission rates. Acad Emerg Med.

[16] Hollander JE, Gatsonis C, Greco EM (2016). Coronary computed tomography angiography versus traditional care: comparison of one-year outcomes and resource use. Ann Emerg Med.

[17] Litt HI, Gatsonis C, Snyder B (2012). CT angiography for safe discharge of patients with possible acute coronary syndromes. N Engl J Med.

